# “We’re Talking About You, Not to You”: Methodological Reflections on Public Health Research With Families With Young Children

**DOI:** 10.1177/1049732320917927

**Published:** 2020-05-12

**Authors:** Rachael Eastham, Alexandra Kaley

**Affiliations:** 1 Lancaster University, Lancaster, United Kingdom

**Keywords:** children, qualitative research, ethics, qualitative interviews, North West England

## Abstract

In this article, we critically reflect upon the experience of public health research involving children and contribute to existing conversations about the methodological and ethical facets of research in this field. Drawing on two phases of a study that sought to explore the lived experiences of families with young children who have had a recent common childhood illness (gastrointestinal infection), we address the research process, from inception of the studies, to fieldwork and the resultant material obtained. We argue that when researching with families about a child-centered experience, it is important to look beyond the individual adult as “participant” and to conceptualize dependents either as, or “like” participants—what we suggest as a “family-centered approach.” Theoretically, this strategy best addresses the lived reality of relationality and responsibility of parent/carers for dependent children; while improving the ease and safety of data collection for the researcher and participants alike.

## Introduction

The lived experiences of children have historically been omitted from child-related health research, which has favored the perspectives of parent/carers or health professionals (Irwin & Johnson, 2005; Morrow & Richards, 1996). It is increasingly acknowledged that children can make valuable contributions to knowledge (Dockett et al., 2009; Morrow & Richards, 1996) and that research should be done collaboratively *with* children, “not on them” (Sammons et al., 2016). Scholars who have experience of working with children assert that children and young people offer unique, specific insights and are both capable and best placed to comment on their everyday lives (Thomson, 2008) and that understandings of their lives would be “fundamentally incomplete,” without their participation (Woodgate & Kristjanson, 1996). However, achieving this inclusivity does not come without its challenges; challenges which explain in part, the tendency for children to be overlooked in research agendas—specifically in respect to the ethical considerations and the practicalities of undertaking research with this age group.

First, with regard to ethical challenges, research with children has frequently been avoided often due to anxieties about the extent to which children lack, or are perceived to lack, the agency to make decisions about their involvement in research and provide informed consent (Dockett et al., 2009; Morrow & Richards, 1996). “Assent,” defined as “the agreement obtained from those not able to enter into a legal contract” (Carter & Ford, 2007, p. 20), is suggested as an alternative for child participants whereby a researcher must ‘attune themselves to the child’s unique communication’ and ‘remain constantly vigilant to the responses of the child at all times’(Cocks, 2007, pp. 258–259 cited by Dockett et al., 2009) to determine if they wish to take part—no mean feat amid the other demands of qualitative research practice such as prompting, probing, and active listening.

(Fear of) risk of harm to the child and preoccupations with child protection may also limit decisions to research with children (Macdonald & Greggans, 2008; Mauthner, 1997; Morrow & Richards, 1996) as children in “Western cultures” may typically be considered a “protected species,” with researchers akin to “predatory adults” (Macdonald & Greggans, 2008, p. 3124). Thus, negotiating with gatekeepers and providing assurances of child safety can be time consuming.

Practically speaking, depending on the age and abilities of the child, research may be undesirable, as children are perceived to lack competencies that would facilitate their participation (Hill, 1996) and are, as such, considered as “objects” rather than valuable research subjects (Mauthner, 1997). Consequently, the “value” that children may meaningfully add to the data collected is routinely questioned and frequently doubted, especially for younger aged children. Securing child-friendly research spaces and designing methods inclusive of children may also be more challenging than arranging meetings with adult participants.

When looking for existing guidance about involving children in research, much of the literature reflects U.K. policy context and conflates “children and young people” despite the huge disparity in capabilities, independence and so on, amid this group (see Alderson, 2011; Crown Copyright, 2014). Although such resources offer very helpful general guidance, there is little available that relates specifically to young children (i.e., aged 7 or younger) (See Sammons et al., 2016, for one exception which refers specifically to these disparities between different aged children and offers a basic figure to help guide research protocol involving children in clinical research).

There is a wealth of literature that demonstrates the range of methods and approaches that can be used successfully when doing research with children (Bryan et al., 2019; Carter & Ford, 2007; Supple et al., 2015; Thomson, 2008); however, there are fewer published studies that depict research designs that have worked less well. Furthermore, although reflexive accounts exist in the literature, particularly by feminist researchers (Morrow & Richards, 1996). Discussions about the practice or “messy dynamics” (Larkin et al., 2014, p. 4) of qualitative research, that is, the “nitty-gritty” detail of how researchers do qualitative research involving children, has tended to be overlooked (Macdonald & Greggans, 2008) and accounts of data collection “sanitized” (Irwin & Johnson, 2005). Macdonald and Greggans (2008) combined both factors—research with children and the practical “reality” of qualitative research in a helpful publication that used case study examples from their own experience of health research to highlight the “chaos and complexity” inherent in the reality of qualitative work with parents and children to reflect on the “pragmatic challenges” (p. 3123).

Therefore, following Macdonald and Greggans (2008) and as a means to extend the discussion by Irwin and Johnson (2005) who wished to “begin the conversation with other researchers” about the challenges of working with young children; this article aims to narrow the gap in the methodological literature of this type. Through critical reflection on the ethical and practical facets of our own family-centered public health research, we contribute reflection points for future researchers who may be planning to work with families with young children and evaluate the (dis)advantages and lessons learned by working with children who are present during research “about them.”

## The Empirical Study

The research upon which this article is based was derived from a qualitative study that was situated within a multidisciplinary research program commissioned by the Health Protection Agency and Public Health England to develop an understanding of the individual and socio-spatial patterning of gastrointestinal infection (GI) and its consequences (see Adams et al., 2018, for existing publications about this project). The qualitative arm of the research sought to explore the lived experience of GIs for families living in socio-economically contrasted neighborhoods in Northern England. We were recruited to undertake this part of the project due to our experience as qualitative public health researchers. Specifically, the researchers are experienced in using creative visual methods as an inclusive strategy to investigate health-related research topics with marginalized young people and adults on “sensitive” issues (e.g., in relation to sexualities, mental health, or disability).

GIs are common. Estimates suggest around 25% of people in the United Kingdom suffer an episode of GI per year (Adams et al., 2018). The main symptoms of GI are diarrhea and vomiting and mild fever. These symptoms usually appear up to a day after becoming infected and typically last less than a week (but can sometimes last longer). While common (and usually short-lived), around 10% of children present to health care services with GI each year, accounting for 16% of pediatric A&E presentations in one study (Armon et al., 2001). In addition, there are 8 million absences from school and at least 11 million working days lost to the economy each year due to GI—thus, demonstrating the burdensome nature of the disease at a public health level. Evidence of the relationship between deprivation and GI suggest that the trends in risk differ between adults and children, with children living in more deprived areas having higher risk of infection and hospitalization (Adams et al., 2018; Tam et al., 2012). However, the mechanisms underlying the relationship between deprivation and GI infection are not well understood, and further work is needed to understand the ways in which interactions within the domestic and public social context increase risk of GI. In addition, there is a distinct lack of qualitative studies that have explored people’s lived experience during a GI infection and how people relate to the places and resources that are available to them locally leading up to, and following an infection.

Given the background statistical information and the qualitative gap in the extant research, our study chose to focus on families with young children aged 0 to 11. This age range was selected because previous studies have identified children of primary school age (and particularly those under the age of 4) at most risk of harm and/or hospitalization because of GI (Lorgelly et al., 2008). Adopting a qualitative visual method-based approach, the specific aims of the study were to examine the ways in which individuals interact with their material, social, and cultural environment to influence the consequences of GI for families living in contrasting socioeconomic neighborhoods. To achieve the stated aims of the research, this study was conducted in two phases.

In Phase 1, the researcher(s) conducted one-to-one semistructured interviews with 21 parent/carer groups with young children (aged 0–11) who had recent GI infection, that is, experienced an infection in the last 6 months. Families were drawn from two socio-economically contrasting areas of a town in Northern England, to draw out any potential differential consequences of GI for families. Recruitment took place via flyers, posters, and face-to-face information distributed at children’s centers, nurseries, primary schools, and toddler groups in the respective areas. To ensure that consent to participate was voluntary and fully informed, potential participants were given the time to decide whether they would like to take part in the study. Full written consent was given at the time of meeting for the interviews—typically 2 weeks after receiving the research project information. These interviews were arranged at participants’ convenience, and often in their homes or at their local children’s center to coincide for ease with other arrangements (e.g., health visitor appointment). The study did not recruit participants whose children were *currently* ill for both ethical and practical reasons. Participants received a £20 gift voucher as a thank you for their participation.

During the interview, participants were invited to take part in a map-making exercise. This was designed to act as a prompt for discussion (Copeland & Agosto, 2012; Crilly et al., 2006) and to elicit place-based information about how the individual had interacted with their social and material environment before, during, and after their illness. This map-making activity ran concurrently to the interview discussion and involved the use of geographical maps, photos, and colored pens, that were affixed by the participants to poster paper.

Phase 2 of the research involved a series of focus groups with adult parent/carers of children aged 0 to 11 who lived in either of the selected case study areas that were a focus for this study. The purpose of this part of the research was to validate our interpretations of the data collated in Phase 1 and to discuss specific themes in greater detail. Focus group participants were recruited via local children centers and through inviting participants who had taken part in Phase 1 interviews. A total of six focus groups were arranged but due to difficulties coordinating times that would best suit entire parent/carer groups, along with many last-minute drop-outs or no-shows (see later discussion), there was a total of nine participants, which meant the sessions were more like semistructured interviews in style. These focus groups took place in children centers local to participant’s homes.

Full ethical approval was gained from the Lancaster University Research Ethics Committee and all participants provided written consent. Each interview and focus group was recorded and transcribed verbatim. The resulting transcript, map/visual material, and fieldnotes were analyzed thematically in Atlas.ti using descriptive, thematic, or theoretical codes relevant to our aim and objectives (Miles & Huberman, 1994). The coding framework was designed by both researchers who conducted the fieldwork; and coding was undertaken by both researchers, to improve validity and reliability. Drawing on the empirical research outlined above, this article involves a discussion about the child-related methodological facets of the research in these two phases. First, we will explain our position and planning around children as considered in the study design phase. Next, a discussion of the children during the fieldwork phase will be presented, followed by reflections on the ethical issues raised throughout the entire process. Excerpts from the researchers fieldnotes are presented to offer deeper insights into the challenges encountered during the research process. In this article, the fieldwork excerpts pertain to different encounters with research participants, all of whom are simply described as “participant” to maintain individual anonymity.

## Conceptualizing “the Participant”

Consistent with the considerations laid out in the background section of this article and our own values of practicing inclusive qualitative research, we discussed at length options for including children as participants in the research process.

Existing ethical guidelines relevant to public health research, for example, those written by the Medical Research Council (MRC, 2004), suggests that research ‘should only include children where the relevant knowledge cannot be obtained by research in adults’(p. 5). After consideration among the research team about the aim and objectives of the GI research project, it was decided that the potential contributions of any children would lie outside the scope of our research aims and objectives. Indeed, given the age range of the children that were the focus of this study, it would not have been practically possible or ethical to include them in the study design as many were of pre-school age (some children were under 1). This observation is based on evidence from the existing literature which recommends that inclusive research *with* children works best when conducted with children aged 7 and above (Bhagat & Howard, 2018; Bryan et al., 2019).

What is more, given that this study sought to understand the consequences of a child’s GI infection for the family as a whole, we perceived that this decision was methodologically and ethically sound and would allow us to obtain the “relevant knowledge” (as set out by the MRC guidelines) needed to answer our research questions.

From this point onwards, we were firmly conceptualizing the individual adult parent/carer as “the participant.” While we did not include children “as participants” in the study design (for the reasons mentioned above), we did acknowledge that the relevant adult participants were to be parents/carers of children aged 0 to 11 years old; and that recruitment was to take place in children’s centers, nurseries, and schools. Given this, we recognized that those recruited would have caring responsibilities and therefore, it would be respectful and appropriate to be inclusive of potential participants by ensuring that their children would be accommodated too.

Our efforts to “account for children” meant arranging spaces in child-friendly sites at child-friendly times to minimize the burden of participation. Prior to recruitment, we met with children’s center staff, other local authority representatives, and an environmental health officer to determine the most appropriate places and ways to recruit participants and conduct the research.

The children’s centers offered among other things, crèche provision, play space, health visitor drop-ins, and community social spaces such as cafes. We were offered rooms in the children’s centers to carry out the research and were invited along to groups to meet parents and explain our research in advance of any interviews. These visits facilitated the majority of our participant recruitment (compared to, for example, flyers handed out to local primary schools); and also provided an opportunity to “get to know” the needs and requirements of the participants and their families.

In Phase 1, the children’s center venue was considered to suitably accommodate the participants who could either: meet with us while their child/ren attended a crèche session; meet with us conveniently when they were due to attend the children’s center for another reason (e.g., health visitor appointment); and/or that we could conduct the interviews in safe child-friendly rooms that included play facilities. We also volunteered to meet with participants in their homes at their convenience. In these instances, the University’s lone working policy was followed, whereby other members of the research team acted as a “buddy” to the fieldworker who logged in and out, before and after each home visit. For the focus group element of the study in Phase 2, it was necessary to conduct the research in community-based, accessible, child-friendly spaces and therefore, children’s center rooms were used to host all these sessions.

Conscious of ethical practice and the wider cultural context of child safeguarding and protection (Parton, 2011) assurances were offered to the ethical review panel, the research gatekeepers (e.g., children center staff) and potential participants in the form of an enhanced criminal records background check obtained by both fieldwork researchers.

However, despite our best efforts in planning how we could best accommodate participants and the children who may accompany them, it transpired that these gestures only went some way to meaningfully consider the needs of those children who were present. With hindsight, the measures taken did little to prepare us for the reality of conducting fieldwork about children, in children’s spaces, with children present; the experience of which will be captured in more detail in the next section.

## The Practical Realities of Fieldwork “About” Children

In Phase 1, despite our “adult only” participant focus, children were present at 11 of the 21 interview sessions. As these interviews took place during the day, Monday to Friday, the children were generally of preschool age and hence at home with their parent/carers. As we expected, these children were not generally of an age or ability where they would have been able to articulate their experiences of the topic in question (in this case, their experience of GI).

The fact that recruitment took place in children’s centers and schools explained the tendency for the participants to be women (*n* = 20/21), as they were often on maternity leave or worked full-time as primary child carers in the home. For this reason, they also had a flexibility of sorts, to arrange to meet and be interviewed. The typical characteristics of the sample group may also explain the attrition rate, especially in Phase 2 focus groups. Where arranging to visit participants at home in Phase 1 was convenient for the participants with caring responsibilities; the inflexibility of the focus group venue outside of the home, limited volunteers’ ability to participate. Many were unable to make it “last-minute” or did not turn up when expected. This significantly reduced the number of participants, with many focus group sessions depleting from an expected five participants to only one. An indication that interviews, with more scope to be flexible to the needs of the individual, may better suit interviewees who have caring responsibilities.

Indeed, our experiences during the recruitment phase of this study highlight the need for a wholly person-centered approach when recruiting participants with any caring responsibilities. This may be especially relevant if research seeks to include women or is women-centered, given how caring for children in British households, continues to be a primarily female occupation (Park et al., 2013). Seeking as much information as appropriate about the likely circumstances during fieldwork meetings (e.g., at our children’s center visits during the recruitment phase) helped the researchers to plan more effectively. Again, despite best efforts, we were often unable to find out much about the participant’s family responsibilities beforehand (how many children, their ages, etc.), which made it difficult to anticipate their needs. Whether the child/ren would be present or not was also subject to abrupt change for example, if the child was off school due to illness, or their child care arrangements had been disrupted. In addition, the specific context could shift the needs of the child and therefore the demands of the interview—for example, if an interview was close to lunch time, or later in the day when a child was tired. It was imperative that the researchers had a flexible, patient and cooperative approach when working with this group. Nonetheless, even with careful planning, the unpredictability of caring for young children meant that the retention of participants throughout the course of the fieldwork process presented a challenge.

Interviewing in the home as in Phase 1 best accommodated their caring responsibilities and subsequently made the meetings much easier for us all—on one occasion, for example, the interview was scheduled to take place during “nap time,” so the participant would be undistracted, while her twins slept in the next room. However, often, upon the researcher’s arrival, the children got excited and interested in the researcher and the research. It soon became apparent how attractive the visual methods and the resources (photos, pens, glue etc.) to explore families’ experiences of GI, was to any small children present. On multiple occasions, especially when the research was taking place around a low table, or on the floor, the children would be grabbing at the pens and pictures, tearing the paper, and generally making a mess. These scenarios were frequently accompanied by troubling reflections for the researchers, as follows in this account about an interview in Phase 1, by Researcher 2:

I arrive at the participant’s home where we had agreed to conduct the research interview. She invites me in and I follow her to the lounge. Her two-year-old son is sitting on the sofa watching the television. I wave hello to him, he looks up clearly interested in my arrival, but then becomes distracted by the television and turns his head back to the screen. I get out the map making materials and lay them out on the coffee table and invite the participant to sit next to me, so we can begin the activity [. . .] her son becomes interested again and comes over to have a look at what we are doing. He watches us work for a while, and evidently becomes a bit bored and agitated and begins pulling at his mum’s sleeve in an attempt to get her attention. She tries to ignore him for a while, until he starts to grab some of the pens and begins drawing on the map that we are making. She takes the pens from him and tells him firmly that he is not allowed to draw on the paper. He doesn’t seem particularly upset by this and continues to grab at the pens and the glue, becoming increasingly excitable. Eventually the boy’s father comes in to the room and takes him upstairs. Following the boy’s absence, the task at hand becomes much easier to complete, and I can talk with the participant more freely and without distraction. I am however, left with a slightly uncomfortable feeling. It seemed inevitable that the child would have wanted to participate in what he perceived to be an “art and craft” activity and his exclusion from the interview, in his own home, seemed a bit unfair.

Of course, with hindsight, this is an entirely predictable behavior, as visual methods are especially useful for work with children who are drawn to images (Thomson, 2008). Whether then it was reasonable or fair to exclude the children from an intrinsically child-friendly research activity, was brought into question on frequent occasions. In contrast, for the adult participant, where the map-making exercise was intended to make the interview process less burdensome by prompting recall and structuring the discussion, for many who needed to multitask, the exercise proved more challenging. On more than one occasion, for example, the participant was breast feeding or trying to pacify/soothe or feed their child while simultaneously attempting to construct a map of their experience.

There was a host of other ways that the dynamic created by children being present impacted the interviews. We shared many of the same experiences reflected in existing accounts of working with small children including “multiple interruptions” (Macdonald & Greggans, 2008, p. 3124) and difficulties creating space to speak directly to the adult participant (Thomson et al., 2012). In our case, the crying, laughter, and chatter; climbing on furniture; and noisy play were complemented with other less predictable events, for example: an interview in Phase 1 which was abruptly halted after a child present vomited into a bin (arguably even more disconcerting during an interview about GI); and a child present in a Phase 2 session, got stuck in a dress-up costume from the crèche area at a children’s center and approached the researcher to help wrestle her out.

During the research meetings challenges also arose regarding the research venues; challenges which were exacerbated by the presence of small children, as reflected in Researcher 1’s fieldnotes from the focus groups in Phase 2:

Upon arrival at the Children’s Centre to host a focus group, I was advised that there had been a double booking and a crèche session was being held in the space originally allocated for my research meeting. The alternative room had some play facilities but was ultimately too small for the 3 adults and 3 children we tried to accommodate. The children played relatively quietly but they were very young and extremely messy! Therefore when, without prior warning, a social worker and her clients arrived for a formal family case review meeting, I was not only flustered by the interruption, but the interview wasn’t complete. The last couple of questions were then rushed through whilst I crawled around cleaning up the toys, attempting to get the room back into a reasonable state to hand over to the group waiting outside. I left feeling unprofessional and very stressed. Fortunately, the participants didn’t mind at all and continued chatting away—much more used to the chaos of these children-related spaces than me.

Such disruptions inevitably interrupted the flow of the interview or focus groups to some degree meaning that the thread of the discussion was lost, or the audio recording was compromised. Indeed, some recordings were so poor due to the noise of children playing and interrupting, they were impossible to transcribe. In these cases, when the meeting had been particularly loud and disrupted, extensive field notes were written immediately to compensate for the potential loss of interview data. These fieldnotes functioned primarily as “a rich source of evidence about the place of the child within the research” (Thomson et al., 2012) prompting reflections about the family dynamic and the ways in which young children influence, and ultimately shape, the domestic space. This was particularly useful when trying to understand the consequences of the GI episode being discussed.

The children’s presence also lingered after the interview. When the sessions were over, there was often an obvious impact left on the space; and certainly, we felt the impact upon ourselves as researchers emotionally, mentally, and physically. After interviewing, especially in the children’s center, it was, of course, our responsibility to tidy up the research materials and to leave the space as we had found it. The children’s center staff were extremely helpful and accommodating and were essentially “doing us a favor” facilitating our recruitment and then supporting fieldwork within their premises. The participants frequently offered to help tidy and clean, but they had the demands of their (by this time often irritable) child(ren) to manage. This “reparation period” proved to be a practically challenging task in both Phase 1 and Phase 2, where the researchers frequently required additional cleaning equipment (hoovers and dustpans) to return the space to its original condition.

This physical labor often exacerbated the emotional and mental fatigue experienced at the end of the interview or focus group. “Standard” interviews, that is, those on a one-to-one basis between adults, can be intense—emotionally laborious and intellectually exhausting as the researcher negotiates competing and simultaneous tasks to ensure a successful research interaction (Dickson-Swift et al., 2007; McGarrol, 2017). In these situations, the “reparation period” exacerbated this fatigue as the next example, from fieldnotes by Researcher 1 from Phase 1, illustrates:

I met the participant with her 18-month old child in a crèche space provided by the children center for an interview in phase 1. I was quite strongly affected by her initial disclosure about previous participation in very sensitive research where she had subsequently been treated in an ethically very dubious way by the researcher. I felt guilty on behalf of “researchers” for her previous encounter. During the interview her child played around us whilst we spoke and was very active, climbing up onto tables and at one point, tipping violently head first into a low set ceramic sink. I was very anxious about all the risks of harm that I observed and felt responsible for distracting her carer. After they left I had much reparation to do: tidying away paper, toys, cleaning pen and paint off a table, resituating furniture—I had certainly not expected to sweep up and replace the contents of a sandpit that day when I had headed to my interview. It took me about 40 minutes and I missed my train—a good lesson for expecting these meetings to take (lots of) extra time.

Despite this circumstance causing no alarm to the parent or child in this interview, the (possible) risk of harm to non-participating children during fieldwork, mentioned in this fieldnote excerpt when the carer was distracted, leads us to consider next, the ethical challenges.

## Talking “About Them”: Ethical Dilemmas in Research About Children

Our original ethical decision making that led to the exclusion of child/ren as participants was called into question most notably in relation to questions about responsibility for child safety and the ethical aspects of relationality and representation in this research. As children had not been conceptualized specifically as “the participant” in this research, despite our efforts to accommodate them and to be as inclusive and helpful as possible, their safety had not been directly considered in the ethical review process. Although we could reasonably assume the spaces were safe for children, as they were either their own homes or approved community-based children-specific sites governed by the necessary health and safety protocols, it became clear that we were not always able to control the research environment, and how children behaved therein. While we “distracted” the adult parent/carer with our research questions children climbed on tables, threw things at each other, wandered off into the crèche toilet spaces; one even partly deconstructed a venetian blind!

This is as important practical lesson regarding “risks of harm” for researchers and ethics committee members to consider. Despite children’s “protected species” status in relation to “predatory” researchers (Macdonald & Greggans, 2008) and the widespread culture around child protection, our efforts to meet the needs of this public were arguably tokenistic, like other versions of public/patient engagement can be by the academy (Hahn et al., 2016; Supple et al., 2015). Overall, any risks of harm were minimal and planning for every eventuality is, of course, impossible. Our intention with this “tokenism” critique in relation to our own work is not to suggest (more) rigid risk assessments and contribute to anxieties about working with children going forwards. On the contrary, we wish to give real examples to better manage expectations, so this type of work is less stressful, and participants’ needs are more meaningfully met. For instance, while the gesture of seeking an enhanced criminal records background check may be welcome especially by gatekeepers and ethics committee, this does little to prevent a child tumbling head-first into a ceramic sink during a research meeting. Being better attuned to the realities could create real benefits for the participant/s and researchers. For example, while meeting in a child safe space like a crèche was helpful and reassuring, offering a simple activity to keep them occupied could have been even better. Thus, we suggest that the focus on “likely risks” imagined during the research design phase could be better aligned with the realities of conducting research with families.

There are also lessons learned on a more theoretical level inspired by the dilemma of responsibility experienced by both researchers during the fieldwork. On one hand, it could be argued that the participant had “chosen” to have their child present and that it was not our responsibility as researchers to ensure that a parent/carer pays “enough” attention to their child. However, we instead understood that the responsibility of caring for a child is not a “choice,” there is after all, no safe option for a parent/carer to do otherwise. Given that, in turn, we were responsible for seeking the views of parents/carers as part our research, acknowledging and accepting a shared responsibility with the parent/carer for the duration of the research meeting, felt like the most ethical standpoint—something which could be achieved in part by meeting the practical needs of the participants in a meaningful ethical way, as suggested in the previous paragraph.

Departing from ethical responsibilities for participant safety and comfort we next consider questions that were raised in relation to representation. One specific example from Phase 1 highlighted the relationality of the parent and child as observed by Researcher 1 and how they are enmeshed (Thomson et al., 2012):

I met the participant in their home, a man (the only male participant so far) whose daughter had been profoundly unwell and hospitalized from an illness that had been initially understood to be GI. This had been very distressing for him, his daughter and his wider family. As we sat on the floor in a room alone and completed the map activity, he called to his partner to get their daughter to come and join in, to tell me what happened and to show me with the pictures on the map. I felt conflicted about the daughter’s participation, after all, we didn’t have ethical approval for children as “participants.” She was shy and said very little but colored onto the maps and helped him stick things on. Their collaboration made me consider the ways that methodologically, we had separated the participant (adult), experience from that of the child in question, when in this case, it was intrinsically shared. Certainly, the participant felt more comfortable being inclusive of their child and not merely speaking “about them” from the next room.

This case resonates with literature in wider social research about the politics and ethics of representation in relation to children (Alldred, 1997) and troubles our assumption that it *was* sufficient for the parent/carer to reflect on the experience on behalf of the child. Indeed, this strategy may have facilitated our collection of “relevant knowledge” about the topic of GI but there was something “bigger” happening when children were present which we had failed to anticipate. During our research, we were often not only privy to personal accounts of GI, but also the social, relational context within which these episodes were lived. The nature of these relationships was indeed part of the “relevant knowledge” in relation to this topic, as this significantly influenced the consequences of GI for the family, as evident in the last example and others experience of distress about the illness of their children—the emotional consequences.

Although stressful in the moment, subsequent reflection on these ethical dilemmas which highlighted issues of responsibility and relationality, were central in our theoretical re-evaluation of the concept of “the participant” in work with families with young children—a key lesson learned which we will discuss further in the next section.

## Expanding Concepts of “the Participant” in Research With Families With Young Children

The reflections in this article relate specifically to research when a key inclusion criterion is that a person has young children where it would be unjust, unreasonable, and exclusionary to request to work with this group only if/when they could guarantee their children would be cared for elsewhere. Indeed, in our research where we sought to contrast experiences between two different socioeconomic areas and the respective consequences, it would have skewed the demographics of the participants to include only people who had the resource (financial or otherwise) to arrange child care. This was our position at the outset of the project which meant we opted to position the individual adult parent/carer as participant while “accounting for children” when planning our research. As we described, this process of accounting for children included among other things: prior consultation with children’s center staff; meetings hosted in child safe spaces with activities to occupy them, for example, play facilities in crèche environments; flexible home meetings; arranging meetings to tie in with visits parents were already making to the children’s centers and so on. To this end, “risks” to children were sufficiently managed from the point of view of ethics.

Nonetheless, we suggest that being inclusive of the children as “like participants” in this research would have enhanced our project including the data collected and would have been the most ethically and methodologically sound option in this instance.

As we have already mentioned caring for a child is not a “choice.” Small children by their “dependent” nature, mean that they cannot be separated from their parents/carers. That is not to say that an adult parent/carer and their child/ren are not “distinctive,” but they are, especially mothers and children “utterly enmeshed” (Thomson et al., 2012, p. 187)—a reality that was very evident during our fieldwork. Thus, the dependent relationality of our research context meant that focusing conceptually on only the individual adult as the participant from the planning stage, challenged the research interaction which in turn may have compromised the data.

(Re)conceptualizing the nature of “the participant” in our research from the outset would have meant we better accommodated the family as whole in this project. If instead we had taken a methodological position that considered child/ren as “like participants” by virtue of their existence and presence, rather than based on their possible “data contribution,” this could have allowed us to better meet needs by reducing the di/stress of the parent/carer and child; limiting the researcher’s dilemma about responsibility in relation to the child’s safety; avoiding the indecipherable audio recordings; and so on.

Our original consideration of doing research “accounting for” small children, that is, by acquiring an enhanced criminal records background check, or using crèche spaces, fell short. If instead, we had used visual activities that had been specifically designed to be inclusive of the children “like participants,” rather than for the adult participant only, we could have safely occupied the child and even maybe created more space for “adult discussion.” Instead of taking a binary “participant” or “non-participant” approach, present children could have occupied a middle-ground, approached “like participants” whereby “participation” relates to practical and ethical matters, rather than research data. This perspective would have meant that the child would have been more meaningfully considered at the research design and ethical review stages (including by the ethical review committee); and may have been better accommodated during the interviews thereby facilitating an easier research interaction with the adult participant.

Informed consent for this “like participant” version of a child’s involvement would be unnecessary if data were not used/collected from them and their “assent” to be included “like participants” would be sufficient. Alternatively, as part of the process and terms of seeking informed consent from a parent/carer, researchers could directly address the presence of their children, their inclusion (or otherwise), and their safety; facilitating better transparency and planning in relation to the needs of the child/ren during the research meeting.

Understanding the parent/carer *and* child as “the participant” could result in a better method choice. In our case as it was, it would have not required much adaptation of our visual activity to create space for small children to “join-in” in some capacity, limiting the tensions between the adult and child present that resulted from the parent/carer attempting to avoid their child “spoiling” their contribution to our research. Alternatively, considering an entire family as the unit of study, in the way of a “case study” could have been most fruitful. Case studies, as an “all-encompassing method” are cited to pay attention to “real-life context” and many “variables of interest” (Yin, 1984, p. 13)—could have presented opportunities to consider the contributions from various family members (participants); and orientated us to collect different types of data that could have informed our understanding of the phenomena.

Also important to note here, is that although children were not included in the research “as participants” they nevertheless had a very active role in shaping our understanding of the phenomena in question throughout the data collection process. Indeed, despite the ethical and practical “challenges” of having children present, their presence also undoubtedly enriched our understanding of the data that we gathered. Given this—we argue that when researching with families about a child-centered experience, it is important to look beyond the individual adult as “participant” and to conceptualize dependents either as, or “like” participants—as a means of recognizing their agency and presence during field work.

While the interview and focus group accounts of the experience and consequences of GI gave us thoughts, feelings, and descriptions in the abstract, the opportunity to observe and participate to some extent, in the interactions between parent/carers and their children deepened our understanding of the lived reality of caring for a child. A lived reality is one which is often “chaotic,” with or without a GI. Although the consensus from our sample was that children’s GI is “just one of those things” the labor involved in their children’s care (obvious as it was during our research meetings—when they were well!) offered us a brief glimpse into the additional daily challenge that a GI episode might present (e.g., having to deal with intense bouts of diarrhea and vomiting) in an already “chaotic” environment. While we, as researchers tasked with collecting qualitative research data, found this environment overwhelming and unpredictable at times, the participants reflected a similar attitude to the study as they did the management of their child’s GI illness, that is, not a big deal. Indeed, their meetings with us as part of this research process did *not* appear to be any more remarkable to them than many of their other day-to-day activities, conducted alongside caring for their children.

Our suggestion is, therefore, that there should be a broadening of the unit of analysis from the participant only to a “family-centered approach,” whereby dependents are considered “like participants.” This is one way we envisage that researchers could be (even) more prepared for the experience of working alongside young children in public health research. While our research study was conducted in the United Kingdom, the experience of common childhood illnesses within the family context is a universal phenomenon, as is the dependency of young children. As such, our “family-centered” approach to public health research may be usefully applied in other countries and cultural contexts.

## Recommendations

As is evident from this commentary, the challenges of working alongside children in a research setting were multiple; however, this created an opportunity to learn for the future and extend the existing evidence base about public health research involving families with small children through the suggestion of a “family-centered research approach,” whereby dependent children (or adults) would be better considered as “like participants,” while many researchers whose work involves children will undoubtably be aware of challenges such as those that are presented in this article and take steps to accommodate the needs of the family appropriately, the difficulties and “messy dynamics” (Larkin et al., 2014, p. 4) are frequently omitted from publications. We hope this article has provided some specific examples to fill this gap, and we now wish to make some direct recommendations for those who may find themselves in a similar fieldwork situation, that is, working on a topic that relates to young children.

First, to stress, we maintain our position from the outset that if a key inclusion criterion for research is “having children,” it is fundamentally unethical to reject children from the research space. It is instead ethical to ensure that the needs of research participants (in this case, individuals with child caring responsibilities) are safeguarded and are not eclipsed by the researcher’s own agenda to obtain certain information in a particular way.

To broaden researchers and ethical committees’ consideration of the “participant” beyond viewing individuals as “sources” of data, we suggest that it may be beneficial to use a “family-centered” research approach and therefore experiment with the conceptualization of “the participant,” being flexible and inclusive as appropriate. Remember that the reality of a research interaction may not align with your theoretical (single) participant. This is especially relevant if research seeks to include parents or is intrinsically family centered. Researchers should seek as much information as possible about the likely circumstances during fieldwork meetings. Explain that you want to know so you can be best placed to do the interview in a way that accommodates the needs of everyone that may be there, while ensuring the participant does not feel under pressure to privilege your needs as a researcher over their own.

In the planning phase when considering the best methods to answer your research questions, consider if you could be more sensitive and inclusive of “the family” as “participant” and be aware that good “data” could be collected in multiple formats, for example, through fieldnotes based on observations of the family relationships. If, for example, your intention is to uphold a rigid, “traditional” interview format, we suggest reconsidering your approach or at least, managing your own expectations in relation to the realities of working with families with small children.

Prior to conducting your research, liaison and consultation with parent/carer groups and/or relevant support professionals in the local area may be helpful in offering place-specific practical guidance on how to accommodate family’s needs during data collection (i.e., where to host research meetings).

Embrace the demands and “messiness” of family life. An adaptable, patient, and compassionate approach is imperative to ensure that this group is enabled to participate in research. If the research is likely to involve the company of small children, having more than one researcher present, especially in focus group settings where this would have less impact on the researcher/participant dynamic, could be very helpful. However, challenging the research meetings may be, focus on the benefits, what the fieldwork is offering rather than what is unachievable. If at all possible, two or more research team members could be present during fieldwork to absorb the demands of research with families of young children, that is, by providing means of “distraction” with age appropriate resources. However, it is also important to avoid assumptions that more people (or more resources) will necessarily make it easier in reality, that is, children may not respond well to the presence of strangers.

Prepare in advance and allocate time to immediately write fieldnotes, especially if/when the audio recording may have been compromised. Allow extra time for reparation and “close down” at the end of the fieldwork session. Not only may the space have been disrupted and as the researcher you might be responsible for rectifying this, but some time for reflection and recovery may be needed.

## Conclusion

The presence of children in both phases of this research project made the fieldwork very challenging from the outset. However, the methodological and ethical lessons learned from the experience have been invaluable in extending our understanding of what it means to take a family-centered approach to research.

Challenges to our audio/transcription-based data were balanced out by the ways that the immersion within the lifeworld of the participant and their child, and observation of their relationship enhanced our understanding of the GI experience. This offered us a flavor of the type of “everyday” demands upon families generally and enriching our understanding of the challenges and burdensome consequences that result when a child had GI and is unable to care for themselves.

This article contributes to the typically lean conversation around the praxeological aspects of doing qualitative research and offers guidance for research involving young children. The “realities” offered here aim to dispel fears about working with this sample group by managing expectations and ultimately, to encourage more safe and fruitful research that is inclusive of families and children.
